# History of the World Allergy Organization: 1989 to 2006, the XVIII World Allergy Congress, Journal Development, Reorganization, and New Programs

**DOI:** 10.1097/WOX.0b013e31822c9540

**Published:** 2011-08-15

**Authors:** Allen P Kaplan

**Affiliations:** 1 Medical University of South Carolina, Clinical Sciences Building, Division of Pulmonology/Allergy

## Abstract

History of the World Allergy Organization: In 1951, the leaders in allergy from all over the world came together to form the International Association of Allergology and Clinical Immunology (IAACI). For the next 60 years, the allergy world converged at the IAACI triennial meetings, which became biennial in 2003. The international meetings, originally named the International Congress of Allergology and Clinical Immunology (ICACI), are now the World Allergy Congress (WAC) hosted by the World Allergy Organization (WAO). Everyone who has aspired to have worldwide recognition has played a part in IAACI-WAO. The History of the WAO traces the global arc of the allergy field over the past 60 years.

The current officers of WAO elected to focus on this rich history, inviting prominent leaders who are interested in being part of this history project to write about their time with IAACI-WAO. This series will be presented in Cancún, México, as part of the XXII World Allergy Congress (December 4-8, 2011). Leading up to the Congress in Cancún, the WAO Journal is presenting segments of the History as part of the "Notes of Allergy Watchers Series." Please enjoy.

--Michael A. Kaliner, MD

Historian, and Past-President (2006-2007),

World Allergy Organization

## 

My time as a Board Member of the International Association of Allergology and Clinical Immunology (IAACI) began in 1989 during the Presidency of Dr Jacques Charpin. Some of the members at that time were Drs S. Gunnar O. Johansson, Alain deWeck, Jack Pepys, Oscar Frick, Albert Oehling, and Michael Kaliner. Its functioning bore little resemblance to the World Allergy Organization (WAO) we all know today, but this was its predecessor, and the organization dates back to 1951. Its origins were primarily European, but the addition of Drs Terumasa Miyamoto from Japan, José Huerta Lopez of México, Leonardo Grieding of Argentina, Julio Croce of Brazil, and Michael Kaliner and myself from the United States, represented the start of a more global distribution of the leadership. Before that time, Dr. Fred Wittich (1981-1984) and Dr. Carl Arbesman (1979-1982) were the only Presidents originating from the US.

A few years passed, and much effort was devoted to the International Congress that was organized every 3 years. This was, in fact, the main task of the Board of Directors because the broad programmatic portfolio we associate with WAO had not yet begun. I was asked to hold the position of Secretary-General in 1992, and although we always had a Treasurer, I signed off on checks for the organization until 2006, when Dr Richard F. Lockey became Treasurer, because it was convenient to have the signatory be someone from the US, given the location of the business headquarters in Milwaukee. It is interesting to note that I have no idea what prompted my choice as Secretary-General or how it transpired. We did not have a formal constitution, there were no elections, and I was not yet a member of the "inner sanctum" responsible for those kinds of decisions.

## Development of the Organization's Journal

One of the next steps the IAACI took was to create a journal. This was the brainchild of Dr Alain deWeck, who created it with the support of Hogrefe and Huber as publishers. It was called *Allergy and Clinical Immunology News*, and was something of a hybrid between a bona fide journal and a magazine. I was appointed an Associate Editor and joined Drs Jean Bousquet and Michael Kaliner. Most of the articles were reviews, but there were some original reports. It had interviews and information about allergology in various parts of the world. It was easy to read, included an occasional cartoon, and was colorful. Dr deWeck remained as Editor-in-Chief for 10 years, and I was an Associate Editor for much of that time. Contributions came from all over the world; thus, it served as a vehicle by which allergists from remote regions, where our specialty was not yet well developed, could participate in something that would unite peoples and we could all meet in-person at the tri-annual Congress.

Many years later, Dr deWeck retired as Editor-in-Chief, and I assumed that position for the next 8 years. We changed the name of the journal to *Allergy and Clinical Immunology International*, but it remained essentially the same, and Drs Kaliner, G. Walter Canonica, and Charles Naspitz served as Associate Editors. However, the number of submissions rose, the number of pages allotted to each issue increased, and most importantly, the number of allergolgists receiving it worldwide increased substantially reaching a peak readership of close to 20,000. It was funded primarily by IAACI itself (and WAO later on), and with the assistance of Dr Ruby Pawankar grants were obtained from UCB, Japan and Taiho Pharmaceuticals. Costs rose as the years passed, and funding became increasingly problematic because the number of individual paid subscriptions covered only a minor portion of the actual cost and we were dependent on our own funds and whatever external support we could get. Once the new name, the WAO became firmly established, the name of the journal was changed once again to the *The Journal of the World Allergy Organization*. I was succeeded as Editor-in-Chief by Dr Johannes Ring.

During those final 2 years of the journal, it was translated into Russian to reach many of the members of the Commonwealth of Independent States (CIS), which represents all of the countries of the former Soviet Union except Estonia, Latvia, and Lithuania. These included a consortium of representatives from Russia, the Ukraine, Belarus, Kazakhstan, Armenia, Georgia, Azerbaijan, Uzbekistan, Turkmenistan, Tajikistan, and Kirgizstan, all under the leadership of Dr Revaz Sepiashvili. The costs for the translation, printing, and distribution were contributed equally by WAO, the CIS Society of Immunology and Allergy, and Dr Lawrence DuBuske's "IRINE" Foundation. Nevertheless, it was decided that WAO should own its journal, and a new online journal was conceived, led by Drs Johannes Ring and Lanny Rosenwasser. Dr Rosenwasser is the current Editor-in-Chief.

## Reorganization and Growth

My time as President began in 2000 and culminated with the World Allergy Congress in Vancouver in 2003. I served as President-Elect during the Presidency of Dr Johansson, and it should be noted that here, too, my position as the next president was somewhat fortuitous, resulting from illness that kept Dr Mario Ricci from serving. He had been selected previously and was the first Vice-President (we had 3 Vice-Presidents at that time), and I was somehow chosen to fill the vacancy.

### New Mission and Name

Dr Johansson's presidency was a time during which the entire international organization and its mission changed. Mr Rick Iber, representing the central office, spearheaded the effort. The vision was to create a society with worldwide representation that was actively involved in education at all levels, supported research where possible, provided assistance to nations trying to develop the specialty of Allergy and Clinical Immunology, and created a communications network with member nations, regional members, and if possible, individual members. We organized councils to oversee each area and committees within each council to carry out the various projects. The name of this organization was changed, employing "The World Allergy Organization/IAACI" for a few years; and then we dropped "IAACI" from the name, but not without some consternation on the part of the membership. There was concern that we would lose the emphasis on Clinical Immunology, but we felt that a less cumbersome and more "catchy" name was needed with "WAO" as the abbreviation. Names notwithstanding, the organization has grown and matured over the years beyond any of our expectations.

The reorganization of WAO started with the creation of councils (Congress, Education, Communication, Specialty and Training, Research) with committees responsible to each one. My role was, in brief, to take all the facets of our reorganization and implement as many as possible within the ensuing 3 years.

### The Launching of the Emerging Societies Program (ESP)

One of the important new programs developed during my presidency was the Emerging Societies Program (ESP), originally conceived by Dr. Connie Katelaris, who was the WAO Treasurer at the time. We invited representatives from regions of the world where we felt we might be able to influence the development of our specialty there, and at the same time, introduce them to WAO.

Our first choice was "Southeast Asia," but we actually covered a broader region extending from Pakistan to Vietnam plus China, Korea, and Japan. The second effort focused on the nations compromising the CIS. We learned a great deal about the state of development of Allergy and Clinical Immunology from invited representatives of each country, catalyzed communication between these nations, and learned how they felt we could be of assistance. Among the requests were pollen traps for both regions; and for the CIS, translating our journal into Russian. We were joined by the American College of Allergy, Asthma, and Immunology (ACAAI) in the ESP endeavor. Since then, the program has expanded to include South America and Central America, and countries representing North Africa and the Arabian Peninsula.

### The Launch of Global Resources in Allergy (GLORIA)

A major educational project that began in 1999-2000, which was one of Dr Johansson's major contributions, was the creation of the Global Resources in Allergy (GLORIA) series of lectures designed to encompass all the major subjects germane to the specialty of Allergy and Clinical Immunology. A series of slides were produced by WAO, and the lectures were offered to member nations on a competitive basis. An application was required; applicants were ranked and accepted to the degree that funds were available. A WAO member would be sent, all expenses paid, so that the person functioned as a visiting professor and could be integrated into whatever national program was scheduled at the time. By the time I left the WAO Board of Directors, about 15 different lectures were available.

## World Allergy Organization Congress XVIII ICACI, Vancouver 2003

**Figure 1:**
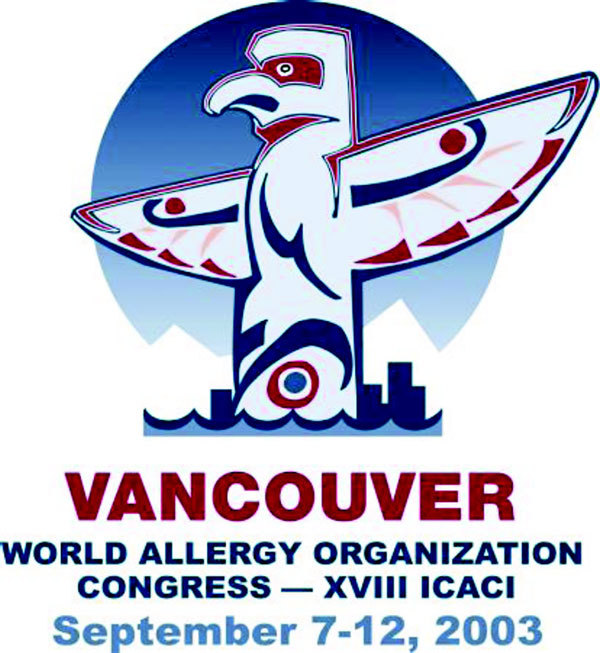
**Meeting logo**.

Certainly a highlight of my Presidency was the meeting in Vancouver in 2003 (Figure 1). The Congress coordinators were Drs Estelle Simons and Michael Kaliner, and together we organized the conference from the choice of venue, negotiations with hotels, development of the program, and so forth. Drs Simons and Kaliner and I conferred by phone on a monthly basis, initially, and then weekly as the meeting date approached. I could not have had more dedicated, efficient, associates to organize this event, and the Secretariat staff members were, as always, critical to its success. The Severe Acute Respiratory Syndrome (SARS) virus hit at about the time of the meeting, particularly in the Toronto area, and may have limited attendance; but we were delighted with the turnout, and the feedback regarding the scientific program and social events was extremely laudatory. We had overestimated hotel rooms needed and did not fill as many as anticipated; however, negotiations between the Secretariat and the hotel managements involved led to a financial compromise such that the meeting was also a financial success.

One event I will not ever forget was the Presidential Dinner. It's a "given" that the food and ambience was great, and we had a full house of invitees including most of my friends (and a few relatives) representing close to 40 years of associations. We had a band and dancing, and I decided to have some fun and participate in the entertainment. In fact, it was rumored among the WAO Board that I had some musical happening planned, but they had no idea what that might be. They did assume that I played some instrument, and a consensus emerged that I was likely to play either the flute or the violin.

I had played the drums between the ages of 4 and 21, though my musical career ended when I graduated Columbia University where I participated in their marching band and concert band. The father of our tympanist was a professional percussionist who was a friend of Lionel Hampton, and Mr Hampton agreed to my having an audition with him, with a view toward my development as a professional jazz drummer. However, one requirement was that I devote full-time to it, that is, give up aspirations of a career in medicine. That did not happen, and after not playing for 40 years, my son gave me a surprise birthday present, 10 lessons with Quentin Baxter who is one of the USAs leading jazz drummers who happens to live in Charleston. I had just begun taking lessons during my presidency, and at the dinner, during the band's break between courses, I arranged to play a drum "solo" with the band's drummer keeping a background beat to complement mine. I cannot know how it really sounded, but the audience response was great; after they recovered from the shock.

## The Last Years on The Wao Board

My last years with WAO as Past-President and then Historian were during the tenures of Drs Carlos Baena-Cagnani and Michael Kaliner as Presidents. However, the duration of both the presidency and interval of the World Allergy Congress was decreased from 3 years to 2 years, so that after 4 years passed, I officially retired from the Board. During Dr G. Walter Canonica's presidency and specifically at the Congress in Buenos Aires last December, I was awarded The WAO Gold Medal (the second recipient, the first being Dr Katelaris), which is one of my most valued honors.

I thought the presidency of the American Academy of Allergy Asthma and Immunology (AAAAI) from 1989 to1990 would be a highlight of my career; and it certainly was, but I also assumed that it would be the last opportunity to contribute to our specialty in association with a major allergy organization. So, my 18-year experience with WAO was unexpected, my ascending to Secretary-General and then President seems like very good luck with the stars aligned just right, and I can honestly say that the opportunity to meet people with like interests from all over the world and to do something helpful for our specialty with worldwide ramifications, is one of the most satisfying things I have ever attempted. The WAO Board members have been a unique, heterogeneous, talented group of physicians to whom I have been indebted for many years because nothing happens without their approval. Many friendships were made along the way that would never otherwise have occurred. The changes from 1998 to 2006 were profound but not formulized until a new constitution was developed under the leadership of Dr Richard Lockey. Standing Committees were added apart from those included within the council structure. The position(s) of Vice-President was abolished, and a mechanism for election of Board Members, Executive Committee members, and the President were established. Democracy arrived; the "old boys" network ended, but there is no progress without loss, and elections mean that there are "losers." It is also a group deception to think that the backroom choices were less effective than the electoral choices, or that popularity contests, and favors cannot also enter into voting. Nothing is perfect.

The WAO is one of the jewels of our specialty, and it was an honor and privilege to be a part of it. I look upon the successes and progress made in the past 2 decades with great pride. We have an endless supply of talent from which to choose, and the future of WAO could not be brighter. I would like to close with a special thanks to 2 persons; Dr Michael Kaliner and Ms Kay Whalen. Dr Kaliner and I began our Allergy Fellowship together in 1970; we worked together at the National Institutes of Health (NIH), overlapped on the AAAAI Board, worked together for WAO during my entire tenure, and have been friends for 40 years. I would probably not have written this had he not asked me to do so. Ms Whalen and I spent 10 years together working for AAAAI and 18 years for WAO. She is understated, sometimes taken for granted, is ever-present, and 28 years of unequivocal support is a long time. I cannot thank her enough.

